# Localized UV emitters on the surface of β-Ga_2_O_3_

**DOI:** 10.1038/s41598-020-76967-6

**Published:** 2020-12-03

**Authors:** Jesse Huso, Matthew D. McCluskey, Yinchuan Yu, Md Minhazul Islam, Farida Selim

**Affiliations:** 1 Klar Scientific, 1615 NE Eastgate Blvd., Unit G, Ste. 3E, Pullman, WA 99163-5300 USA; 2 Department of Physics and Astronomy, Washington State University, Pullman, WA 99164-2814 USA; 3 Department of Physics, Bowling Green State University, Bowling Green, OH 43403 USA

**Keywords:** Semiconductors, Electronic devices

## Abstract

Monoclinic gallium oxide (β-Ga_2_O_3_) is attracting intense focus as a material for power electronics, thanks to its ultra-wide bandgap (4.5–4.8 eV) and ability to be easily doped *n*-type. Because the holes self-trap, the band-edge luminescence is weak; hence, β-Ga_2_O_3_ has not been regarded as a promising material for light emission. In this work, optical and structural imaging methods revealed the presence of localized surface defects that emit in the near-UV (3.27 eV, 380 nm) when excited by sub-bandgap light. The PL emission of these centers is extremely bright—50 times brighter than that of single-crystal ZnO, a direct-gap semiconductor that has been touted as an active material for UV devices.

## Introduction

Monoclinic gallium oxide (β-Ga_2_O_3_) is a potentially important material for power electronics applications, owing to its ultra-wide bandgap and ability to be doped *n*-type^1–6^. In contrast to nitride semiconductors, Ga_2_O_3_ bulk single crystals can be grown from the melt without the need for extreme pressures. β-Ga_2_O_3_ (space group *C*2/*m*) is the thermodynamically stable phase, with **a** ⊥ **c**, **b** ⊥ **c**, and an angle of 104° between the **a** and **c** axes^7,8^. (Hereafter, β-Ga_2_O_3_ is referred to as Ga_2_O_3_). Point defects affect properties such as electrical conductivity and the critical breakdown field^9^. Along with altering the electronic properties, defects are responsible for UV/visible emission bands in photoluminescence (PL) and cathodoluminescence (CL) experiments.

Density functional calculations indicate that the valence band is very flat, the conduction-band minimum (CBM) is at the Brillouin zone center, and the bandgap is 4.5–4.8 eV^10–12^. A photo-generated hole in the VB will form a self-trapped hole (STH) that is localized at a specific oxygen atom^13–15^. An electron in the CB, on the other hand, is delocalized and acts as a free electron. The intrinsic recombination of electrons with STHs results in the UV band, peaked at 3.4 eV (360 nm)^16–18^. Because the self-trapping involves significant lattice relaxation, this emission is rather weak. Bands at lower energies, designated UVʹ (3.1 eV, 400 nm), blue (2.9 eV, 430 nm), and green (2.4 eV, 520 nm), are due to donor–acceptor pair transitions^19–24^.

Annealing Ga_2_O_3_ in hydrogen is a potential method to passivate defects and improve material quality. Prior work has shown that hydrogen forms complexes with gallium vacancies^25^, acceptor dopants^26,27^, and possibly iridium^28^. By removing compensating defect levels, hydrogen passivation may reduce ion impurity scattering and hence improve free-electron mobility. However, hydrogen reduction can cause oxide crystals to decompose. In this work, we report PL emission correlated with surface pits in hydrogenated Ga_2_O_3_.

## Methods

In the present work, undoped β-Ga_2_O_3_ bulk single crystals were grown at Tamura Inc., Japan by the edge-defined film-fed growth (EFG) method. The samples were diced to 5 × 5 × 0.5 mm and annealed in hydrogen atmosphere in closed ampoule at 950 °C. X-ray diffraction (XRD) measurements of the as-grown and hydrogenated samples showed that the crystals are oriented in the (010) direction and the of degree crystallinity improved after hydrogen annealing (Supplementary Fig. S1). This result is qualitatively similar to prior work on thin films, which showed that annealing in air increases the degree of crystallinity and surface roughness^29^. Optical transmission spectra showed a bandgap onset at 4.5 eV (Supplementary Fig. S2).

PL and PL excitation (PLE) spectra were obtained with a Horiba FL3-21 Spectrofluorometer. A Xe lamp and monochromator were the excitation source, and emitted light was detected by a monochromator and photomultiplier tube. For PLE measurements, the PL intensity at a specific wavelength (380 nm) was measured as a function of excitation wavelength. These PL and PLE measurements were not spatially resolved and therefore give average quantities. To investigate this PL emission with submicron resolution, a PL map was generated with a Klar Mini Pro UV microscope equipped with a 355 nm CW laser and Ocean Insight Maya2000 Pro spectrometer.

## Results

**Figure 1:**
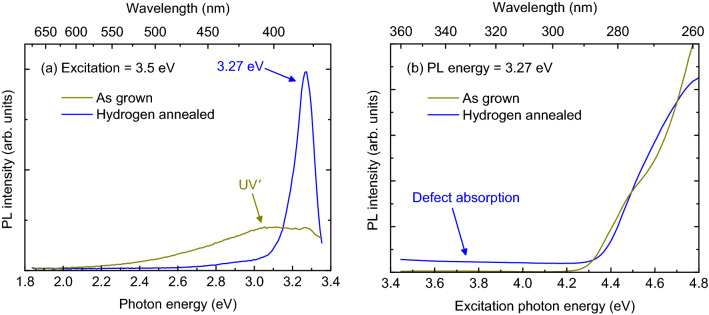
(a) Room temperature PL spectrum of as-grown and hydrogen-annealed Ga_2_O_3_ under 355 nm (3.5 eV) excitation. The 3.27 eV peak and UV’ band are indicated. (**b**) PLE spectrum of the PL intensity at 380 nm as a function of excitation photon energy. The sub-bandgap defect absorption, which gives rise to the 380 nm (3.27 eV) peak, is indicated.

PL spectra with 355 nm (3.5 eV) excitation are shown in Fig. 1a for the reference and hydrogenated sample. The reference sample shows the UVʹ band at 400 nm. A smaller peak is observed at 380 nm (3.27 eV). Because this peak occurs well below the UV band, it is attributed to a defect. In the hydrogen-annealed sample, the 3.27 eV peak is large and clearly dominant. From this we conclude that hydrogenation increases the density of the defects responsible for the 3.27 eV peak.

Additional insight can be gleaned from the PLE spectrum for 3.27 eV photon emission (Fig. 1b). For the reference sample, the major contribution to the PL intensity comes from the UVʹ band. The PLE spectrum shows an absorption onset at 4.4 eV, slightly below the bandgap of 4.5 eV observed with optical transmission spectroscopy. At excitations > 4.7 eV, the PLE increases due to growth in the UV band. Below the bandgap, the PLE intensity is very low.

The hydrogenated sample also has a PLE onset due to the UVʹ band, although the exact PLE profile is slightly different than the as-grown sample. The PLE spectrum does not show an increase from the UV band at photon energies above the bandgap, presumably because defects compete with the intrinsic PL. The disappearance of the shoulder at ~ 4.4 eV may be due to hydrogen passivation of defects.

Importantly, the hydrogenated sample shows significant PLE intensity *below* the bandgap. The shape of the 3.27 eV PL peak does not depend on the excitation photon energy. The sub-bandgap signal arises from the defect that absorbs photon energies > 3.4 eV and then emits at 3.27 eV. Due to experimental limitations, we could not examine the PLE spectrum < 3.4 eV. Therefore, the difference between the emission peak and absorption threshold (Stokes shift) is < 0.13 eV.

We now turn to the spatial distribution of the 3.27 eV emission. The hydrogen-annealed sample was mapped at a spatial resolution of 700 nm, for a total of 1.8 × 10^7^ spatial (*x*, *y*) points, while the reference was mapped at a spatial resolution of 600 nm for a total of 2.7 × 10^6^ points. The integration time was 10 ms per point. The 3.27 eV peak was fit using a bi-Gaussian function using GPU acceleration.

**Figure 2:**
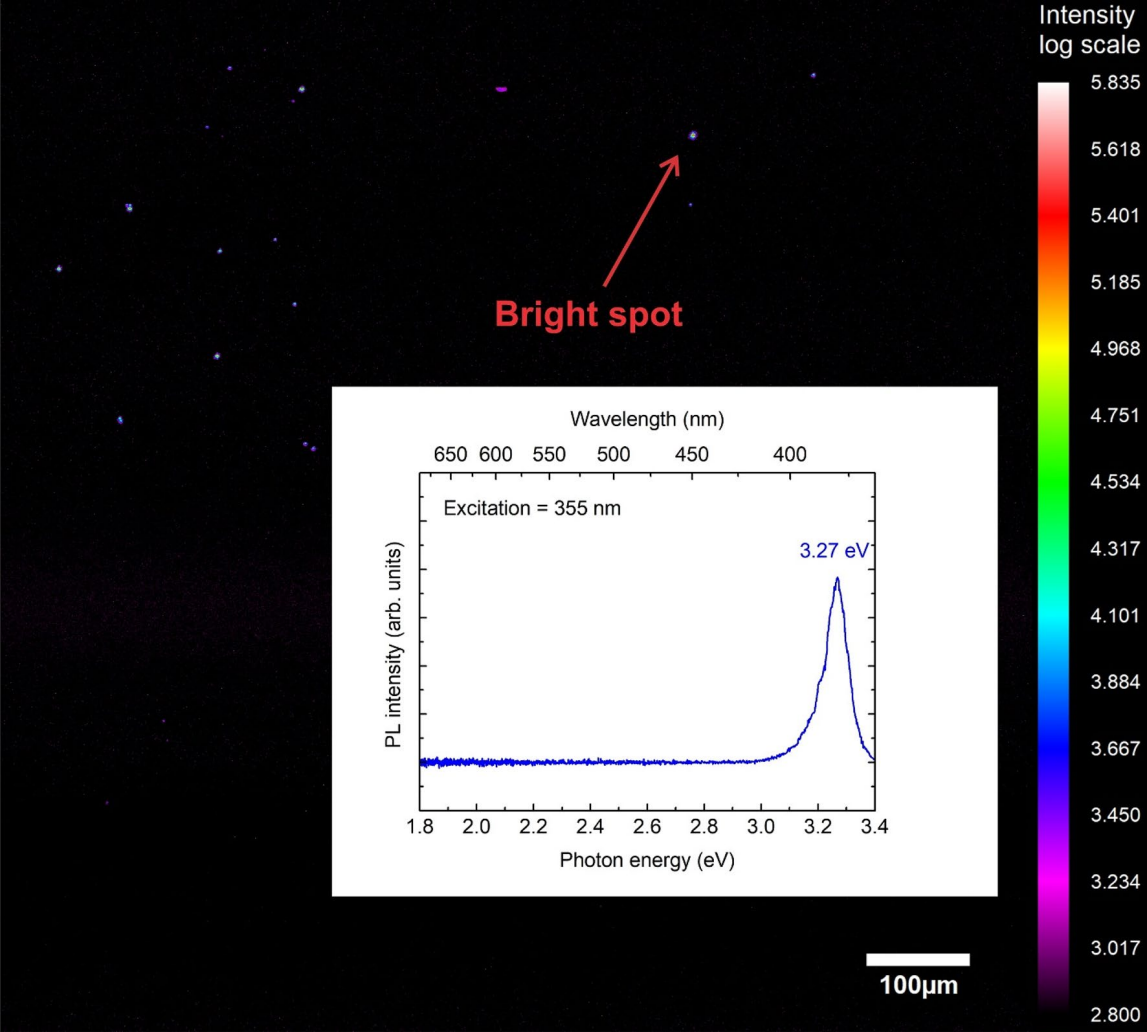
Map of the PL intensity of the 3.27 eV peak for the reference Ga_2_O_3_ sample. Inset: PL spectrum of one of the bright spots.

**Figure 3:**
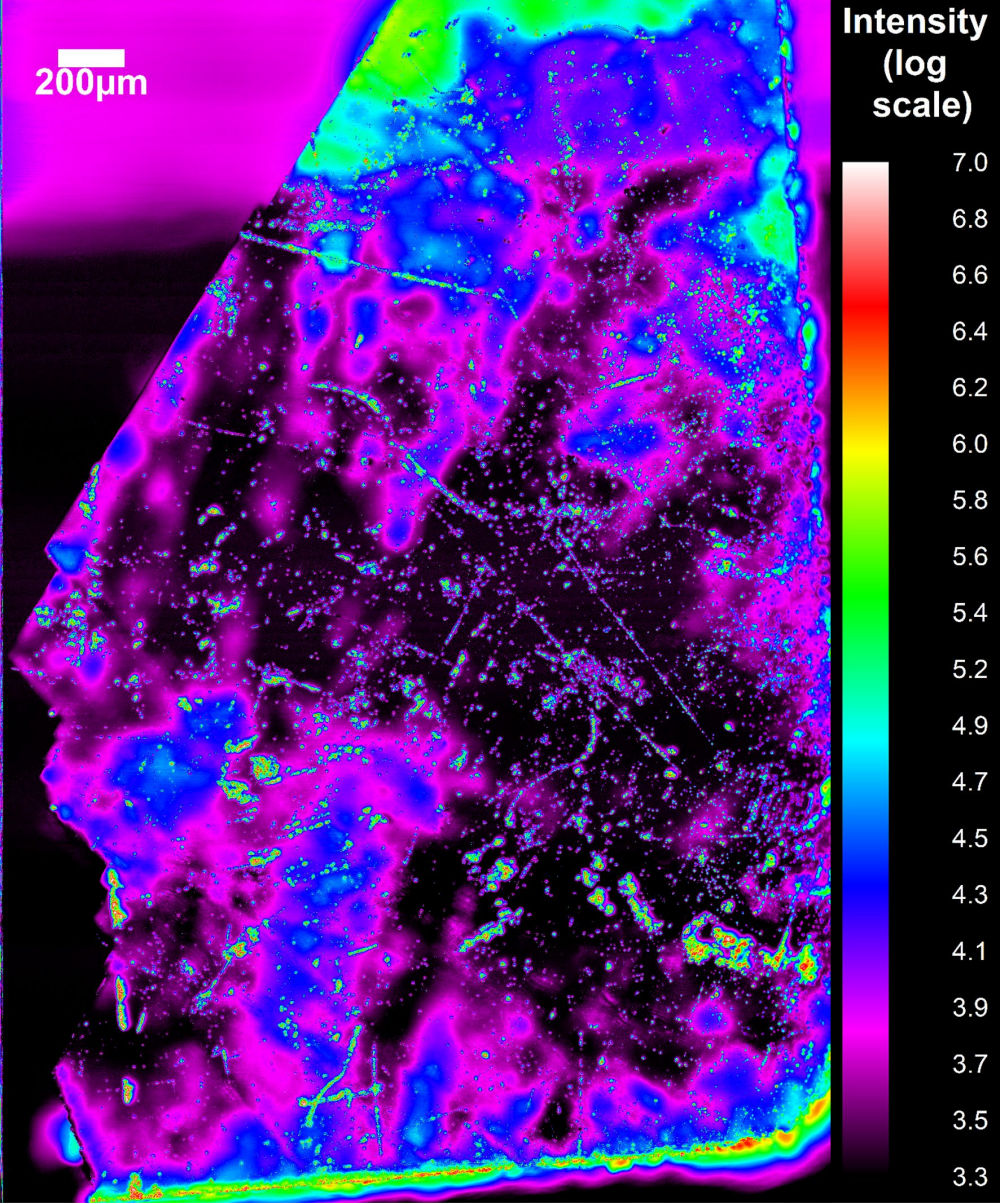
Map of the PL intensity of the 3.27 eV peak for the hydrogenated Ga_2_O_3_ sample.

For the reference sample, the PL intensity was essentially zero except for a few distinct spots (Fig. 2). These localized emission centers showed the characteristic defect emission at 3.27 eV. The hydrogenated sample had a much higher areal density of UV emitters, consistent with the spatially averaged PL/PLE results. Figure 3 shows a PL map of the entire hydrogenated sample. Here, weak defect emission was observed everywhere, but especially bright regions were detected as well. The 3.27 eV peak in the spatially averaged spectrum (Fig. 1a) is primarily due to these localized emission centers.

**Figure 4:**
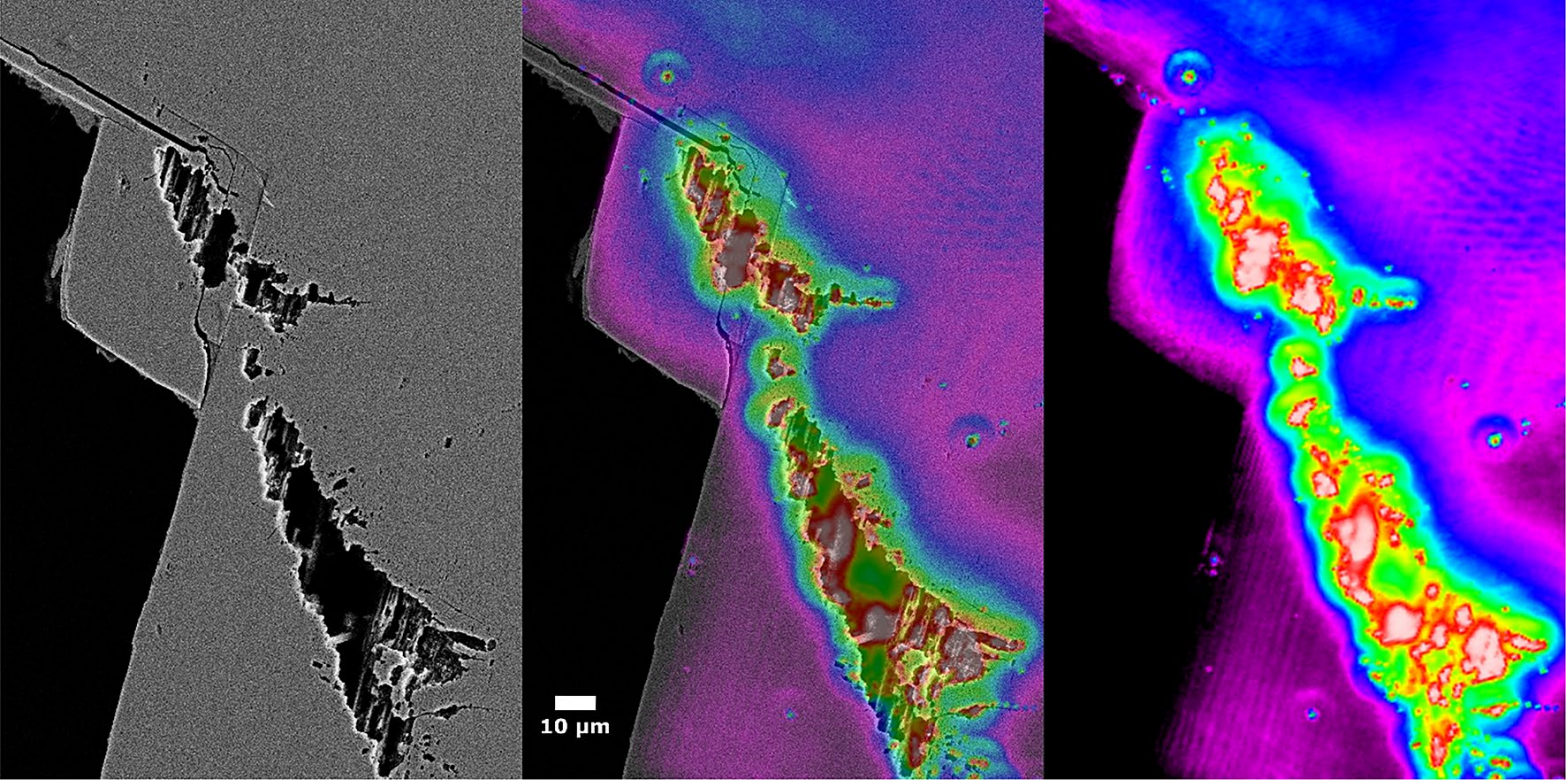
SEM image (left) and map of the 3.27 eV PL intensity (right). An overlaid image is in the center. The defect emission intensity strongly correlates with the surface pits.

To see where these bright emitters come from, we took a scanning electron microscope (SEM) image and compared it to the PL map of the same area (Fig. 4). The SEM image shows surface pits that were caused by hydrogenation. The long axes of the surface pits are aligned along the **c** direction. As shown in Fig. 4, there is a clear correlation between the surface defects and defect emission intensity.

**Figure 5:**
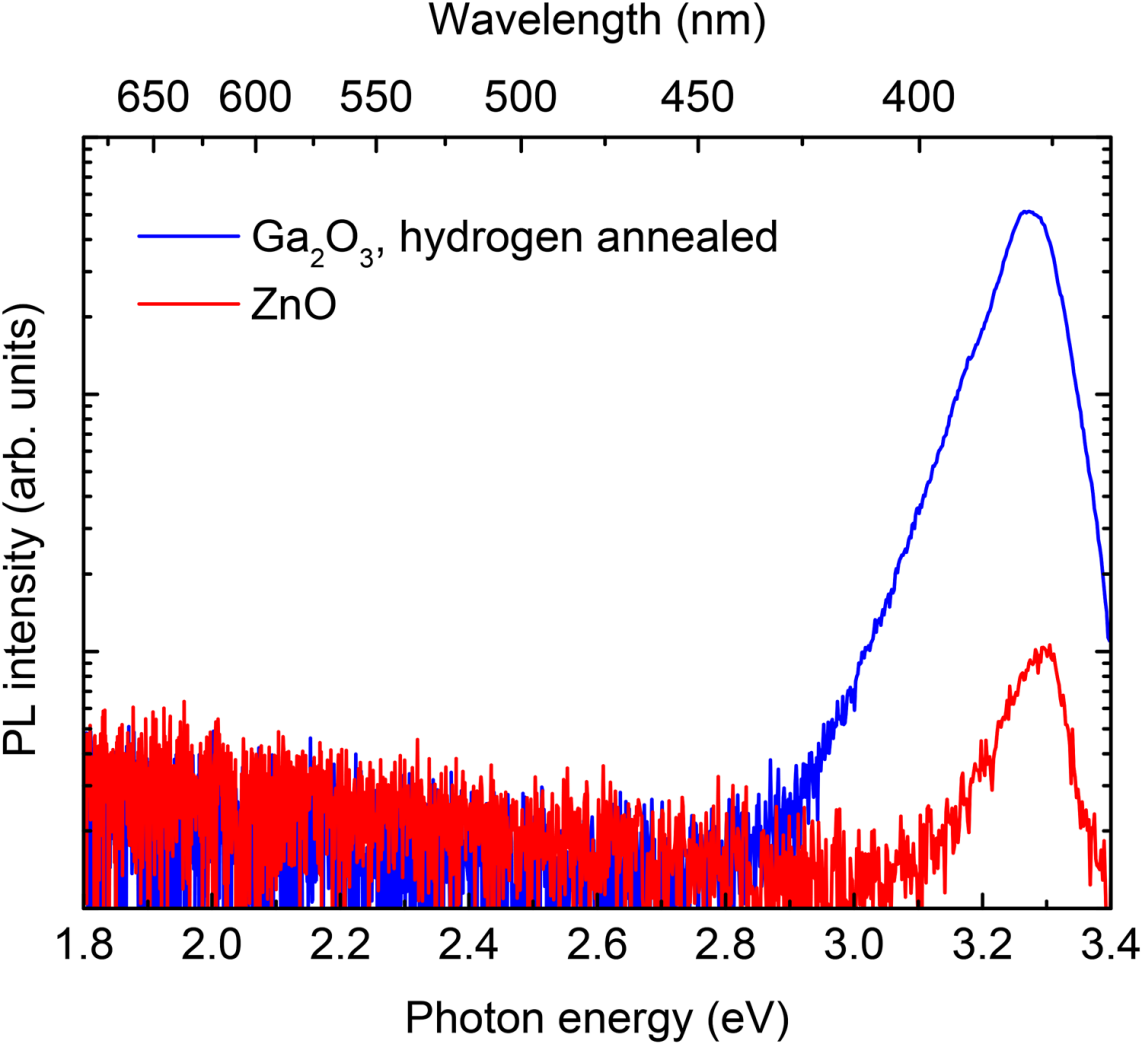
PL spectra (log scale) from an emitter on the surface of hydrogen-annealed Ga_2_O_3_, and bulk ZnO. The experimental conditions were 1 mW laser power and 10 ms integration time. The PL peak height for Ga_2_O_3_ is approximately 50 times that of ZnO.

Some emitters are so bright, low laser power (< 0.6 mW delivered to the sample) and short integration times (10 ms) had to be used to avoid saturating the spectrometer. The PL intensity versus laser power was linear over the measured range (Supplementary Fig. S3). To obtain a useful benchmark comparison, we collected PL maps of hydrogenated Ga_2_O_3_ and hydrothermal bulk ZnO under the same experimental conditions. The similarity between the emission energies of these two materials facilitated the comparison. Both samples showed near-UV peaks and no other peaks down to 700 nm (Fig. 5). The lack of defect peaks in ZnO (e.g., the yellow Li-related band^30,31^) is due to the high excitation laser intensity, which increases the quantum efficiency of the near-UV emission at the expense of defect emission^32^.

Figure 4 shows that the Ga_2_O_3_ emitters have an intensity *50 times* that of ZnO. Prior work measured an external quantum efficiency of 0.5% for bulk ZnO at room temperature and low excitation intensities (5 W/cm^2^)^33^. Assuming the 50:1 ratio applies at low excitation power, the quantum efficiency of the Ga_2_O_3_ emitters is ~ 25%. This relatively high value is consistent with the lack of an observed Stokes shift, which suggests a transition that does not involve significant lattice relaxation^34^. For example, an electron may transition from the valence band to a defect level. If the defect does not relax, then energy is conserved by phonon emission by the hole. After the hole reaches the top of the valence band, the electron and hole recombine, creating a photon.

What is the identity of these bright, localized emitters? Prior work has shown that hydrogen diffuses into Ga_2_O_3_ and increases its conductivity^35^. Annealing in a reducing atmosphere such as hydrogen also decomposes the surface and may result in Ga-rich pits. Elemental mapping of the pits does show evidence that the pits have an excess of Ga and also Si (Supplementary Fig. S4). It is plausible that the pits have a high population of surface or near-surface defects, which emit at 3.27 eV. Alternatively, the pit surfaces may have “intrinsic” surface states that result in the 3.27 eV peak. Regardless of their microscopic structure, these centers are strongly correlated with the surface pits. It is conceivable that these pits act as cavities that enhance the PL intensity.

## Conclusions

In conclusion, bright, localized near-UV (3.27 eV at room temperature) emission was observed on the surface of Ga_2_O_3_. The emission centers show efficient PL emission when excited by photons with energies above 3.4 eV. The brightness of these emitters is remarkable given the generally weak luminescence observed in this material. The strongest emission occurs near surface pits, which are created by annealing in hydrogen. We note that typical PL measurements average over a large spatial region. The results of this study provide a compelling case that one cannot assume that emission centers are distributed homogeneously throughout Ga_2_O_3_. Rather, the bright emission centers observed here occur only at specific, localized regions on the surface. These localized emitters are reminiscent of those found in 2D semiconductors^36^, which also involve defects that have not been positively identified. Future research will improve knowledge of such centers and potentially harness them for optoelectronic or quantum technologies.

## Supplementary information

Supplementary Information.
