# Supplementary material for: Localized UV emitters on the surface of β-Ga2O3
Source: Sci Rep. 2020 Dec 3;10:21022. doi: 10.1038/s41598-020-76967-6 (PMC7712825; doi:10.1038/s41598-020-76967-6)
Supplement: Supplementary file 1 — Supplementary Information. [file 41598_2020_76967_MOESM1_ESM.pdf]

Localized UV emitters on the surface of Ga_2O_3 – Supplementary material

Jesse Huso, Matthew D. McCluskey, Yinchuan Yu, Md. Minhazul Islam, Farida Selim

X-ray diffraction

X-ray diffraction (XRD) spectroscopy was performed on the as-grown and H-diffused β-Ga_2O_3 single crystals using Rigaku diffractometer. The samples were irradiated by K_α line of Cu and the K_β line was minimized using a β filter (Ni). The intensity of diffracted X-ray was recorded as a function of angle 2θ from 10 deg to 90 deg at a scanning speed of 2 deg/min. A broad background from the mount was subtracted from the spectra. The sample exhibits a peak at 2θ = 60.36° (FWHM= 0.809 ± 0.011), which corresponds to the reflection from (020) plane of β-Ga_2O_3. The crystals show highly crystalline β-Ga_2O_3 phase with monoclinic space group C2/m symmetry. The H-diffused sample spectrum shows a sharp XRD peak, indicating high crystalline quality, superimposed on a much weaker broad peak. The broad peak may be due to the defective near-surface layer.

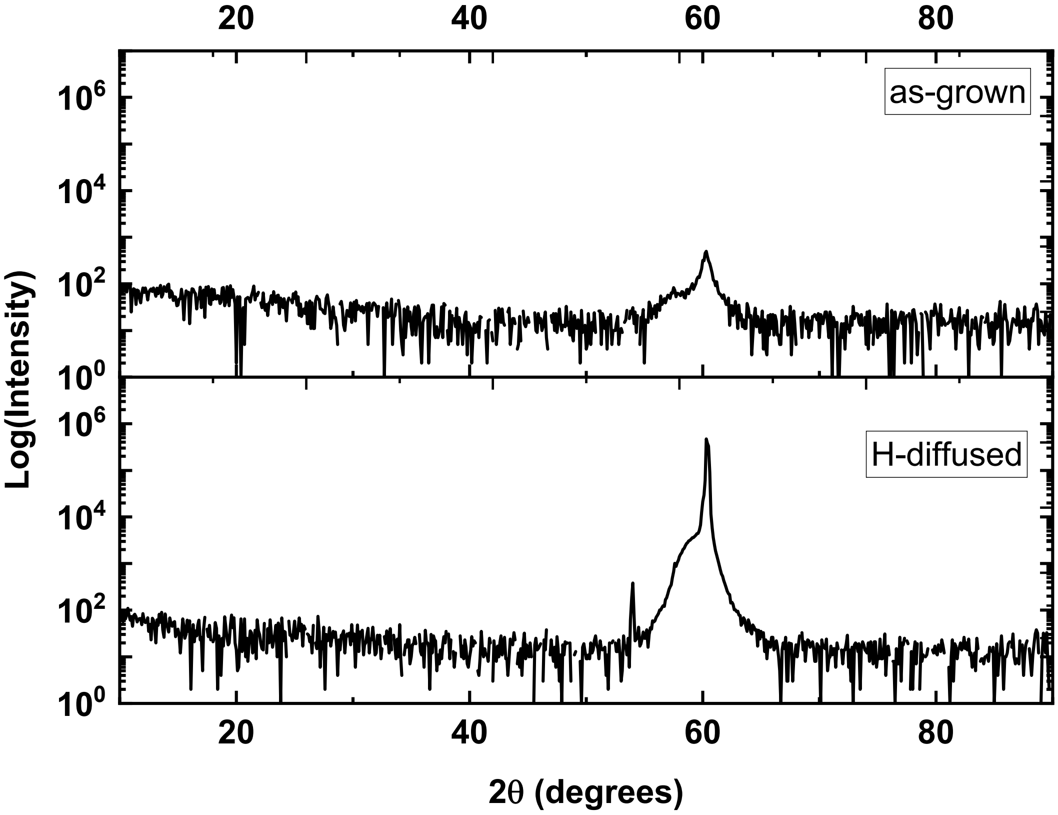

Fig. 1. XRD of as-grown and hydrogen-annealed single crystal Ga_2O_3.

Optical transmission

UV-Vis absorption spectroscopy was performed on the as-grown sample to determine its optical bandgap. The absorption spectrum was recorded at room temperature using a PerkinElmer ultraviolet–visible–near infrared (UV Vis-NIR) spectrometer covering from 1100 nm to 190 nm. The scan speed and slit width for the experiments were set as 240 nm/min and 1 nm, respectively. β-Ga_2O_3 is transparent up to UV region of electromagnetic radiation due to having a wide energy bandgap of 4.4-5 eV.[1-4] The energy of the absorption edge was found to be different in the directions parallel and perpendicular to the b-axis of the crystal due to the anisotropy.[4,5] Moreover, experimental reports have shown that the bandgap determined this way is smaller for single crystals (4.52-4.6 eV) than for films (4.75-5.0 eV).[4-6]

Light was incident normal to the (010) sample surface. The sample showed an energy cut-off at around 270 nm with a shoulder at around 300 nm. This shoulder arises from the anisotropy of optical absorption in β-Ga_2O_3 for light polarized along the *a* and *c* axes.[4,5] The bandgap of the sample is found to be approximately 4.49 eV calculated from $(\alpha h\nu)^2$ vs hν plot (Tauc plot), where $\alpha = 2.303\frac{A}{t}$ is the absorption coefficient, *t* is the thickness of the sample, *h* is Planck's constant and ν is frequency.[3,7]

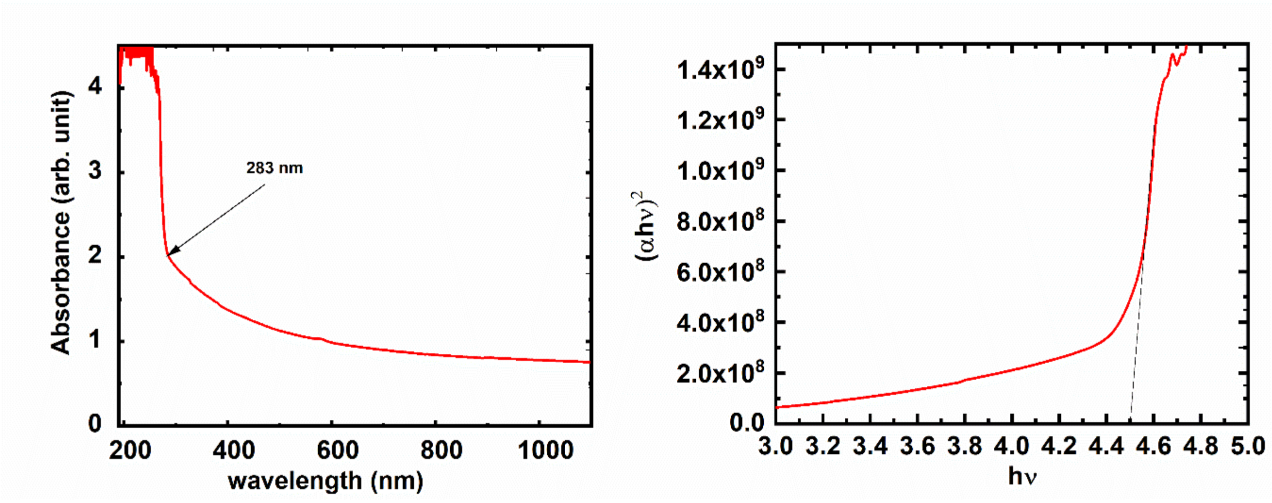

Fig. 2. Absorbance and Tauc plot derived from optical transmission spectra of Ga_2O_3.

Laser excitation power

The laser power was varied from 1.0 to 5.0 mW, which resulted in a power of 0.28 to 1.39 mW on the sample. The PL spectra are nearly the same, with a slight shift (-0.01 eV) and broadening at high power. The peak intensity of the PL is reasonably linear versus excitation power.

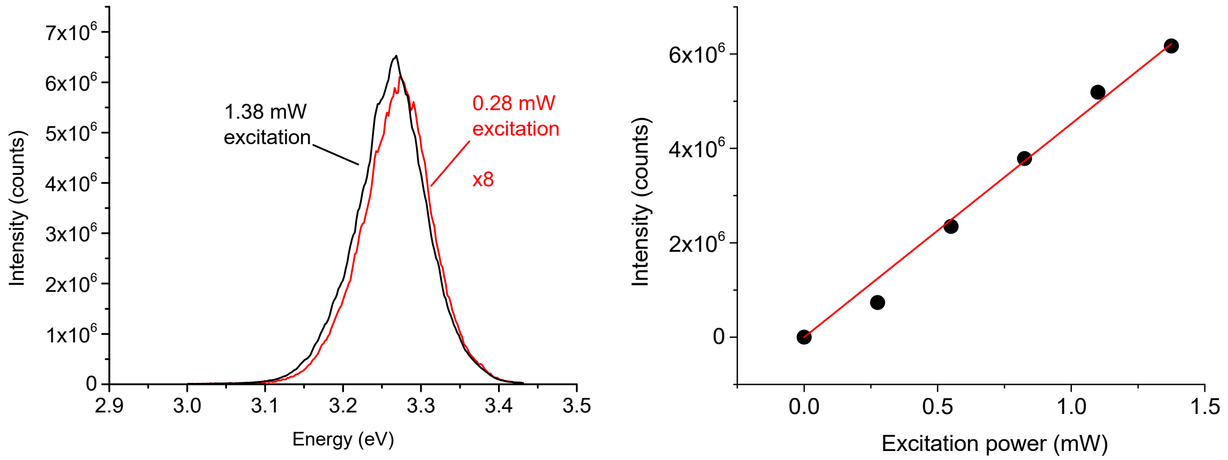

Fig. 3. Left: PL spectra for 0.28 and 1.38 mW laser power on the sample. The 0.28 mW spectrum was multiplied by a factor of 8. Right: PL peak intensity versus laser power. The straight line is a linear fit to the data with the intercept fixed at zero.

Scanning electron microscopy (SEM)

The same region was scanned with an SEM and PL microscope. The PL emission correlates with surface pits on the Ga_2O_3 surface, although not all regions of the pits show emission. Energy dispersive spectroscopy (EDS) shows evidence for excess Ga and Si in the emitting regions.

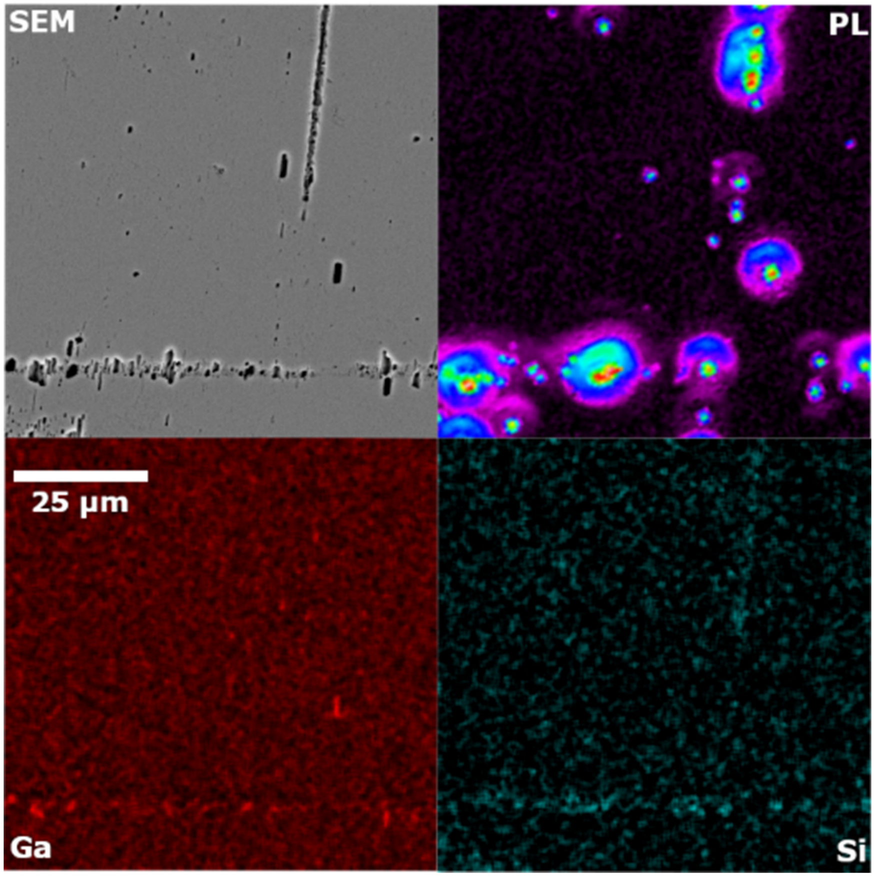

Fig. 4. SEM, PL, and elemental mapping (Ga and Si) of a hydrogen-annealed Ga_2O_3 sample.

References

[1]S.J. Pearton, J. Yang, P.H. Cary, F. Ren, J. Kim, M.J. Tadjer, and M.A. Mastro, Applied Physics Reviews **5**, 011301 (2018).

[2]M. Higashiwaki, A. Kuramata, H. Murakami, and Y. Kumagai, J. Phys. D: Appl. Phys. **50**, 333002 (2017).

[3]M.M. Islam, D. Rana, A. Hernandez, M. Haseman, and F.A. Selim, Journal of Applied Physics **125**, 055701 (2019).

[4]T. Onuma, S. Saito, K. Sasaki, T. Masui, T. Yamaguchi, T. Honda, and M. Higashiwaki, Jpn. J. Appl. Phys. **54**, 112601 (2015).

[5]N. Ueda, H. Hosono, R. Waseda, and H. Kawazoe, Appl. Phys. Lett. **71**, 933 (1997).

[6]M. Orita, H. Ohta, M. Hirano, and H. Hosono, Appl. Phys. Lett. **77**, 4166 (2000).

[7]M.S. Haseman, P. Saadatkia, J.T. Warfield, J. Lawrence, A. Hernandez, G.E. Jellison, L.A. Boatner, and F.A. Selim, Journal of Elec Materi **47**, 1497 (2018).
